# Myeloid control of dendritic cell priming after mechanical HIFU in colorectal cancer: implications for PD-1/PD-L1 blockade

**DOI:** 10.3389/fimmu.2026.1998296

**Published:** 2026-09-28

**Authors:** Yao Liao, Xiyue Yang, Xiaobo Du

**Affiliations:** 1 Sichuan Clinical Research Center for Radiation and Therapy, Mianyang Central Hospital, School of Medicine, University of Electronic Science and Technology of China, Mianyang, China; 2 Department of Oncology, Mianyang Central Hospital,School of Medicine, University of Electronic Science and Technology of China, Mianyang, China

**Keywords:** colorectal cancer, dendritic cells, immune checkpoint blockade, macrophages, mechanical HIFU, neutrophils

## Abstract

Mechanical high-intensity focused ultrasound releases tumor antigens and danger signals. It provides a potential substrate for immune checkpoint blockade. However, antigen release does not necessarily generate productive dendritic cell priming. In colorectal cancer, this transition occurs within a mucosal microenvironment shaped by epithelial barrier integrity, microbial exposure and myeloid clearance. Neutrophils can support epithelial repair or reinforce tumor-associated suppression. Macrophages remove apoptotic granulocytes and regulate inflammatory resolution. They can also restrain dendritic cell cytokine production. These context-dependent functions may determine whether mechanically released antigens initiate cytotoxic immunity or enter a nonproductive clearance response. This mini review integrates intestinal myeloid homeostasis with experimental evidence for mechanical ultrasound ablation and PD-1/PD-L1 blockade. It distinguishes antigen uptake from cross-presentation, protective resolution from tumor-permissive suppression, and colorectal evidence from observations in other tumor models. Functional dendritic cell readouts, lineage-resolved checkpoint measurements and concurrent barrier assessment provide a framework for evaluating combination therapy. The therapeutic objective is to preserve mucosal protection while sustaining antigen-specific immunity.

## 1 Introduction

Immune checkpoint blockade has transformed treatment for mismatch-repair-deficient colorectal cancer (CRC). Early pembrolizumab studies linked response to mismatch-repair status (1), and KEYNOTE-177 established clinical benefit in microsatellite-instability-high advanced disease (2). Nevertheless, this benefit cannot be generalized to mismatch-repair-proficient tumors. Accordingly, the principal translational population considered here is microsatellite-stable/mismatch-repair-proficient (MSS/pMMR) CRC, while dMMR/MSI-H disease is treated as a distinct checkpoint-responsive setting. Local tumor destruction and immune activation therefore need to be considered as distinct therapeutic events.

High-intensity focused ultrasound (HIFU) offers a means of generating tumor-derived antigenic material. Its immunological consequences depend on the mode of tissue injury and the cells that process the resulting debris. In CRC, neutrophils, macrophages and dendritic cells (DCs) also maintain an epithelial surface exposed to microorganisms. A treatment that enhances tumor inflammation while disrupting this protective organization may fail to produce durable immunity. The relevant sequence extends from antigen release through myeloid processing to checkpoint-sensitive T-cell activation.

Recent advances in spatial and single-cell immunology have further emphasized that colorectal cancer immunity is organized by specialized cellular niches rather than by tumor antigenicity alone. Intestinal macrophages are continuously replenished from circulating monocytes and undergo context-dependent functional adaptation to preserve epithelial homeostasis while responding to tissue damage (3). This balance is particularly relevant for therapeutic strategies that intentionally induce tumor injury, because inflammatory amplification and effective antigen-specific immunity are not interchangeable outcomes. Macrophage-derived regulatory programs, including IL-10-dependent signaling, contribute to intestinal immune tolerance and demonstrate why myeloid suppression cannot be interpreted without considering anatomical context (4). Neutrophils provide another layer of complexity. Their recruitment can promote epithelial repair through localized oxygen regulation and hypoxia-inducible factor signaling, whereas unresolved granulocyte activation may sustain pathological inflammation (5). The subsequent clearance of apoptotic neutrophils by phagocytes links innate cell turnover with regulation of IL-23/IL-17-driven inflammatory circuits (6). Therefore, the myeloid compartment surrounding a mechanically disrupted tumor represents an active decision-making environment that determines whether released antigens are converted into productive adaptive immunity or absorbed into a tissue-repair program. In colorectal cancer, this distinction is further shaped by epithelial barrier integrity and microbial exposure. Classical intestinal dendritic cell subsets differ in their capacity for lymphatic migration and antigen presentation, indicating that antigen localization alone cannot predict immune activation (7). Barrier disruption and microbial-derived signals can also reinforce IL-23/IL-17-associated tumor-supportive inflammation, creating a context in which additional tissue damage may have divergent immunological consequences (8). Fusobacterium nucleatum and other microbiota-associated factors further influence the composition of the tumor immune microenvironment, highlighting the unique challenges of mucosal tumors.

This mini review aims to examine how mechanical HIFU-induced tumor disruption is interpreted by the mucosal myeloid ecosystem of colorectal cancer. We focus on the transition from antigen release to dendritic cell licensing, with particular attention to neutrophil–macrophage coordination, epithelial barrier preservation and the conditions required for effective PD-1/PD-L1 blockade. By integrating evidence from HIFU studies, intestinal immune homeostasis and tumor immunology, we discuss a model for evaluating when myeloid responses support rather than restrict antitumor immunity. To avoid conflating these settings, we distinguish direct CRC evidence after mechanical HIFU, CRC or intestinal evidence obtained without HIFU, and evidence extrapolated from other tumor or inflammatory models; links not directly tested in mucosal CRC are treated as hypotheses. Primary mucosal tumors, subcutaneous experimental CRC and metastatic lesions are considered separately because their immune and anatomical contexts are not interchangeable.

## 2 Mucosal myeloid organization defines the antigen context

### 2.1 Protective clearance and granulocyte–phagocyte feedback

Intestinal macrophages maintain a dynamic relationship with recruited monocytes. Adult mouse studies demonstrate continual monocyte replenishment of much of this compartment (3). Macrophage IL-10 receptor signaling restrains spontaneous colitis (4), indicating that anti-inflammatory programming is an essential protective function rather than an intrinsically undesirable state. Its therapeutic removal requires a tissue-specific rationale.

Neutrophils also contribute to resolution. Their respiratory activity consumes oxygen and supports epithelial hypoxia-inducible factor responses during mucosal inflammation (5). After neutrophil apoptosis, ingestion by macrophages and DCs reduces IL-23-dependent IL-17 production and granulopoietic drive (6). This feedback connects granulocyte recruitment to mononuclear phagocyte clearance. Persistent inflammation can therefore reflect failure of coordinated resolution, not simply excessive abundance of one lineage.

This coordinated response also clarifies why tissue repair and antitumor immunity cannot be ranked on a single inflammatory scale. Epithelial protection may coexist with reduced inflammatory signaling, while tumor-associated suppression can occur within an intensely inflamed lesion. Neutrophil oxygen consumption and phagocyte feedback demonstrate different routes toward resolution (5, 6). The biological outcome depends on which neighboring cells receive the signal and whether the epithelial barrier remains functional.

### 2.2 Barrier failure converts protective sensing into tumor support

Antigen sampling and adaptive priming are functionally separable. Intestinal CD103-positive DCs migrate in lymph, whereas CX3CR1-positive sampling cells largely remain tissue associated (7). In intestinal tumors, barrier defects expose myeloid cells to microbial products and promote an IL-23/IL-17 growth-supporting circuit (8). *Fusobacterium nucleatum* can further increase intestinal tumorigenesis and recruit tumor-infiltrating myeloid populations (9). Mechanically released tumor antigens thus enter a preconditioned mucosal niche rather than an immunologically empty compartment.

These observations define two different consequences of microbial sensing. A contained response supports local defense and clearance. Sustained exposure within a disrupted tumor barrier can instead maintain growth-supporting myeloid signaling (8, 9). Ultrasound-induced damage may intersect with either state, depending on the treated compartment. Tumor-associated inflammation and adjacent mucosal inflammation therefore require separate interpretation. Increased inflammatory transcription is not, by itself, evidence of improved tumor antigen presentation.

## 3 Mechanical HIFU releases antigens but does not guarantee priming

### 3.1 Ablation modality determines the material available to myeloid cells

Thermal HIFU produces coagulative injury, whereas mechanical HIFU emphasizes physical tissue disruption. In subcutaneous MC38 tumors, mechanical treatment enhanced DC recruitment and tumor-specific immune responses (10). Mechanical and thermal protocols also generated distinct debris in an ovalbumin-expressing lymphoma model; stronger antigen-specific T-cell proliferation was demonstrated with mechanical treatment combined with CpG (11). This adjuvant contribution should not be attributed to ultrasound alone.

Boiling histotripsy uses boiling-induced bubbles to produce mechanical disruption. It should therefore not be described as entirely free of thermal initiation. Experiments including CT26 tumors support its immunogenic potential, but the anti-PD-1 combination arm was performed in 4T1 breast cancer (12). Separately, nonthermal histotripsy enhanced checkpoint therapy using anti-CTLA-4 in noncolorectal models (13). These studies support a physical ablation–immunity connection without establishing interchangeable mechanisms across modalities or checkpoint targets.

Debris quality is also shaped by exposure geometry. Mechanical HIFU experiments demonstrate that tissue disintegration and immune stimulation vary with the applied protocol (11). Comparisons should account for the fraction destroyed, residual viable tissue and accompanying heating. Otherwise, stronger inflammation could reflect a larger injury rather than a distinct immune mechanism. Sonodynamic therapy and ultrasound-assisted drug delivery should not be treated as equivalent evidence for mechanically ablative HIFU.

### 3.2 Antigen routing and dendritic cell licensing are separate steps

Antigen delivery to draining lymph nodes does not require one universal transport route. After boiling histotripsy of fluorescent-antigen-expressing melanoma, findings supported predominantly cell-free lymphatic antigen delivery followed by uptake by several nodal populations (14). BATF3-dependent conventional type 1 DCs (cDC1s) provide specialized cross-presentation capacity (15). Increased fluorescent uptake or CD86 expression alone nevertheless does not establish peptide–MHC-I presentation and productive priming of naive CD8+ T cells.

Innate licensing provides another requirement. STING-dependent cytosolic DNA sensing supports recognition of immunogenic tumors (16), but extracellular DNA after HIFU is not proof of STING activation in DCs. CCR7-bearing DCs can transport tumor antigen and support priming in melanoma (17); this route must be distinguished experimentally from passive drainage. Both antigen availability and receiver-cell competence need to be measured.

This distinction has practical consequences for antigen tracking. Fluorescent tumor material within a macrophage identifies uptake, not transfer to a cross-presenting DC. Likewise, nodal accumulation does not show that the antigen was processed into a relevant peptide. BATF3-dependent cross-presentation provides a functional reference (15). Matched measurements of antigen-bearing cells, peptide presentation and naive T-cell proliferation can determine where the post-ablation sequence succeeds or fails.

Antigen identity adds a further mucosal constraint. Barrier disruption can expose immune cells to microbial material as well as tumor debris (8). An increased T-cell response after ablation should therefore be tested for tumor specificity rather than interpreted from total activation alone. Defined tumor-antigen assays and recognition of viable target cells can separate antitumor priming from unrelated inflammatory activation. This distinction becomes especially important when comparing mucosal tumors with nonmucosal implantation sites.

Direct combination evidence is encouraging but remains context dependent. Mechanical HIFU combined with anti-PD-L1 improved local and distant tumor control in breast cancer models and altered macrophage programs (18). As shown in **Table 1**, colorectal antigen-release evidence and checkpoint-combination evidence currently arise from partly different experimental systems. Their intersection requires direct validation in mucosal CRC. Accordingly, the integrated HIFU-myeloid-DC-T-cell pathway is presented here as a testable framework rather than an established mechanism in mucosal CRC.

**Table 1:** Experimental evidence connecting mechanical ultrasound ablation to antigen handling and checkpoint therapy.

Model/evidence	Intervention	Immune observation	Interpretive boundary	Reference
Subcutaneous MC38 CRC	Mechanical versus thermal HIFU	DC recruitment and tumor-specific immunity	No checkpoint arm; nonmucosal implantation	(10)
EG7 lymphoma	Mechanical HIFU with CpG	Enhanced antigen-specific T-cell proliferation	Adjuvant-dependent combination effect	(11)
Subcutaneous CT26 CRC	Boiling histotripsy versus thermal HIFU	DAMP release, DC maturation and activated CD8+ T-cell infiltration	No anti-PD-1 combination arm in CRC	(12)
4T1 breast cancer	Boiling histotripsy plus anti-PD-1	Enhanced checkpoint-combination activity and antitumor CD8+ T-cell responses	Checkpoint arm was breast cancer, not CRC	(12)
B16F10 melanoma	Boiling histotripsy	Nodal antigen uptake and DC activation	Cell-free drainage; uptake is not cross-presentation	(14)
Murine breast tumors	Mechanical HIFU plus anti-PD-L1	Local/distant control; myeloid remodeling	Not intestinal CRC or a clinical trial	(18)

CRC, colorectal cancer; DC, dendritic cell; HIFU, high-intensity focused ultrasound. All studies listed are preclinical.

## 4 Neutrophil–macrophage crosstalk regulates productive immunity

### 4.1 Dendritic cell function links antigen release to checkpoint sensitivity

BATF3-dependent DCs are required for effective anti-PD-1 and anti-CD137 immunotherapy in experimental tumors (19). During anti-PD-1 treatment, T-cell-derived IFN-γ and DC-derived IL-12 form a reciprocal activation circuit (20). DCs therefore support both initiation and maintenance of cytotoxic immunity. A large antigen pulse cannot compensate automatically for a dysfunctional cytokine relay.

Checkpoint expression also requires cellular attribution. DC-specific PD-L1 deletion enhanced antitumor T-cell immunity in experimental models (21). Mechanical HIFU increased PD-L1 on granulocytes, macrophages and DCs in breast tumors (18), but this observation does not identify the therapeutically decisive lineage. Bulk PD-L1 staining should consequently be paired with DC state and antigen-specific function.

### 4.2 Neutrophil recruitment can impose suppression beyond PD-1

In KRAS-driven CRC models, reduced IRF2 activity increases CXCL3-dependent recruitment of CXCR2-positive myeloid-derived suppressor cells; pathway inhibition improves anti-PD-1 activity (22). These populations should not be equated with all neutrophils. Colon-cancer-associated neutrophils can activate TGF-β through matrix metalloproteinases and suppress infiltrating T cells (23). Such effects provide a route by which a granulocyte-rich niche can constrain the output of otherwise successful antigen priming.

Tumor-induced neutrophil extracellular traps (NETs) can shield malignant cells from cytotoxic lymphocytes (24). This is an effector-access mechanism, not direct evidence that NETs prevent DC cross-presentation. After HIFU, increased neutrophil numbers or extracellular DNA alone cannot establish either process. NET-associated structures, functional suppression and antigen-specific killing need separate assessment.

The suppressive phenotype also needs functional confirmation. CXCR2-directed recruitment and neutrophil-mediated TGF-β activation were established through different experimental approaches (22, 23). They should not be collapsed into a single universal granulocyte program. A post-HIFU sample may contain recruited antimicrobial cells alongside suppressive cells. Their relative contribution is better resolved by lineage phenotyping coupled to T-cell suppression assays than by a blood neutrophil count or a broad myeloid marker.

### 4.3 Macrophage clearance balances resolution and immune restraint

Macrophages can suppress the DC cytokine relay. In mammary tumors, macrophage-derived IL-10 reduced DC IL-12 and limited chemotherapy-associated CD8+ T-cell responses (25). MerTK blockade also enhanced macrophage STING activation through tumor-derived cGAMP and P2X7R-dependent signaling in experimental tumors (26). These findings identify potentially modifiable clearance-associated programs, but neither establishes a validated MerTK or IL-10 intervention after HIFU in mucosal CRC.

Clearance efficiency and inflammatory output are not equivalent measurements. A macrophage can remove apoptotic cells while simultaneously changing the cytokines available to neighboring DCs. The neutrophil-clearance feedback and the macrophage IL-10–DC IL-12 circuit illustrate these separable functions (6, 25). Their coexistence after HIFU is plausible but remains unproven. Demonstrating antigen retention in macrophages would still be insufficient to conclude that these cells competitively deprive cDC1s of antigen.

The protective counterpart must remain visible. Clearing apoptotic neutrophils restrains granulopoietic feedback (6), while macrophage IL-10 responsiveness protects intestinal homeostasis (4). Antigen-presenting neutrophil states have also been identified in tumors (27), arguing against indiscriminate granulocyte depletion. As shown in **Figure 1**, the relevant distinction is between coordinated clearance that preserves barrier function and suppressive signaling that interrupts DC–T-cell cooperation. **Figure 1** therefore uses graphical evidence coding to distinguish CRC observations from relationships extrapolated from other models and from links that remain hypothetical in mucosal CRC.

**Figure 1:**
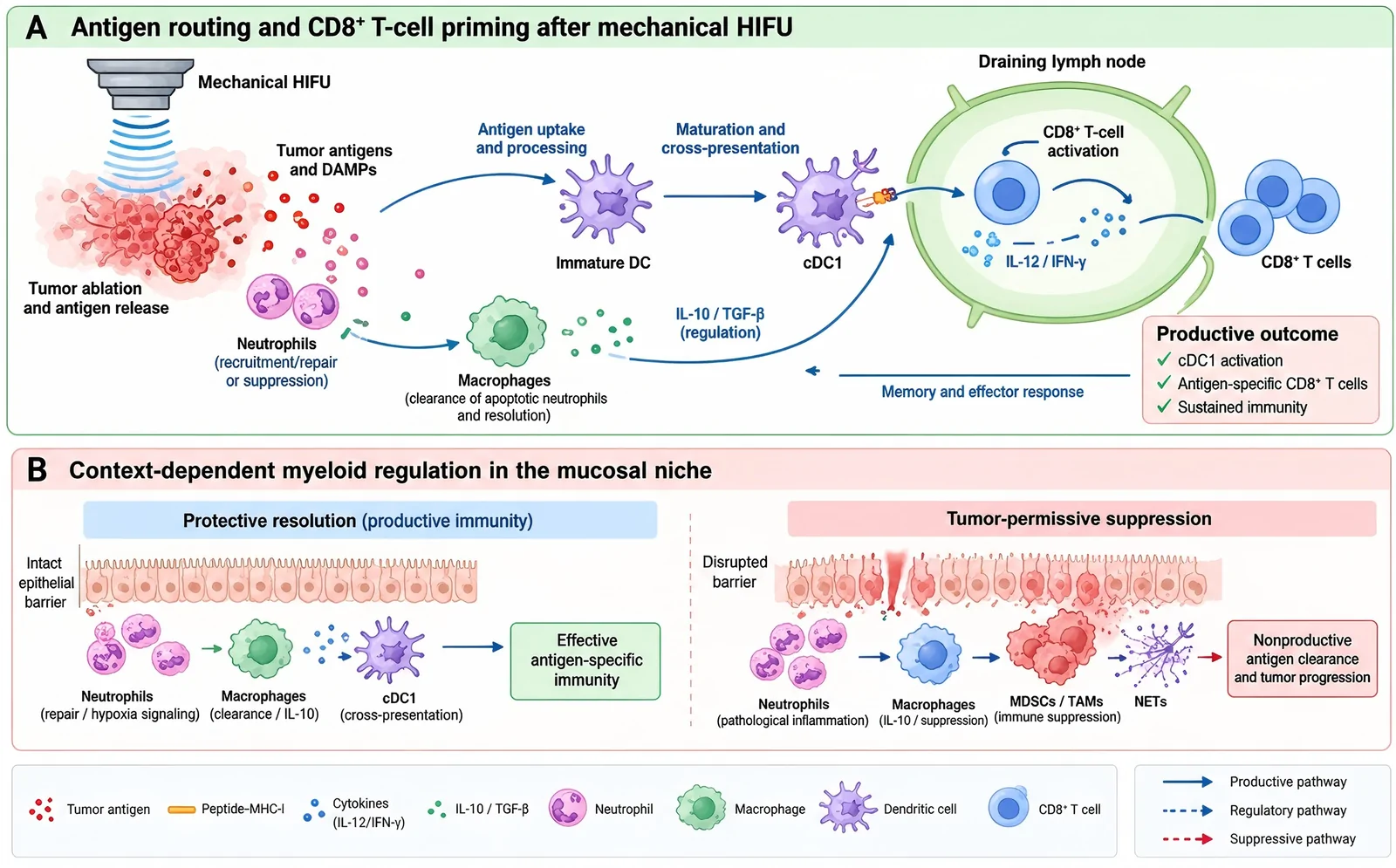
Evidence-stratified antigen routing and Evidence stratified antigen routing and myeloid regulation of CD8+ T-cell priming after mechanical HIFU in colorectal cancer. **(A)** Mechanical HIFU releases tumor antigens and DAMPs, followed by antigen uptake, DC maturation and cross-presentation, and antigen-specific CD8+ T-cell activation. Neutrophil- and macrophage-mediated repair, clearance, and regulatory signals may influence whether this sequence results in productive and sustained antitumor immunity. **(B)** Context dependent myeloid regulation in the colorectal mucosal niche. An intact epithelial barrier supports coordinated neutrophil–macrophage resolution and cDC1-mediated antigen-specific immunity, whereas barrier disruption may favor pathological inflammation, MDSC/TAM- and NET-associated suppression, and nonproductive antigen clearance.

## 5 Context-dependent strategies for combination therapy

### 5.1 Couple checkpoint release to functional myeloid responses

Combination design should distinguish an antigen deficit from a licensing deficit. When antigen-bearing DCs retain IL-12 competence and support antigen-specific T cells, PD-1/PD-L1 blockade has a mechanistic substrate (20, 21). When CXCR2-associated suppression dominates, selective pathway modulation has a preclinical CRC rationale (22). Adding broad myeloid depletion to every HIFU regimen would ignore these distinct failure modes. The treatment objective is restoration of the interrupted function rather than maximal leukocyte removal.

The anatomical stage of failure is equally important. Poor nodal priming requires a different intervention from effective priming followed by impaired tumor-cell access. NET-mediated shielding illustrates the latter situation (24). Anti-PD-1 treatment can reinforce a functioning DC–T-cell cytokine circuit (20), but it should not be assumed to reverse every physical or cytokine-mediated barrier. Combination experiments should therefore retain separate endpoints for priming, tumor infiltration and cytotoxic function.

### 5.2 Preserve epithelial protection during immune reprogramming

Mucosal safety is a mechanistic endpoint. NETs impaired intestinal barrier function in experimental colitis, and their degradation reduced injury (28); these findings do not establish treatment of HIFU-associated or checkpoint-associated colitis. Conversely, protective neutrophil–epithelial signaling and macrophage IL-10 responsiveness argue against indiscriminate blockade (4, 5). As shown in **Table 2**, antitumor readouts should be interpreted alongside epithelial injury and resolution. No validated biomarker threshold currently emerges from these studies for selecting a clinical post-HIFU regimen.

**Table 2:** Candidate research endpoints for testing myeloid-guided HIFU combinations in CRC.

Compartment	Candidate research endpoint	Mechanistic interpretation	Boundary/safeguard	Reference
Tumor/draining node	Antigen-bearing cDC1; peptide–MHC-I; CD8+ priming	Separates antigen uptake from productive presentation	CD86 alone is insufficient	(14, 15)
DC–T-cell niche	DC IL-12; T-cell IFN-γ; lineage-resolved PD-L1	Tests a checkpoint-responsive cytokine relay	Not a validated clinical biomarker or selection threshold	(20, 21)
Granulocyte-rich tumor	CXCR2-associated cells; active TGF-β; NET structures	Identifies recruitment or effector-stage suppression	Demonstrate function, not markers alone	(22–24)
Macrophages/adjacent mucosa	Apoptotic-neutrophil uptake; epithelial integrity	Distinguishes resolution from persistent injury	Preserve barrier-protective responses	(4–6, 28)

cDC1, conventional type 1 dendritic cell; NET, neutrophil extracellular trap. All measurements are candidate research endpoints and have not been validated as clinical biomarkers or treatment-selection thresholds.

Local selectivity may be more important than complete pathway inhibition. Macrophage IL-10 signaling supports intestinal protection (4), whereas macrophage-derived IL-10 can restrain DC activity in a different tumor setting (25). This apparent contrast reflects differences in cellular receiver and tissue context. It supports testing restricted exposure and reversible modulation rather than presuming that systemic suppression of a resolving pathway will improve the therapeutic balance.

## 6 Discussion and future directions

### 6.1 Restore mucosal context in experimental models

Human CRC contains spatially organized immune hubs, including myeloid-associated inflammation at damaged tumor–luminal interfaces (29). Subcutaneous tumors do not recreate this topology. Orthotopic or autochthonous CRC models should therefore compare treated tumor, draining node and adjacent mucosa. Mismatch-repair status, baseline inflammation and microbial exposure should be documented separately. Their contributions cannot be inferred from the tumor cell-line name alone. Accordingly, primary mucosal CRC, subcutaneous models and metastatic lesions should be reported and interpreted as distinct settings rather than pooled under a single CRC category.

Emerging DC-based approaches reinforce the importance of recruitment. A recent study linked mechanical HIFU-induced CCL19/21 signaling to enhanced antigen-loaded DC vaccine activity (30). This is not evidence that endogenous cDC1 recruitment alone explains checkpoint synergy. Causal experiments should combine lineage-specific perturbation with antigen tracking, DC cytokine measurements and functional T-cell assays. Preserved tumor killing after removal of the proposed mediator would argue against that mediator being essential.

Ablation alone, checkpoint blockade alone and their combination require direct comparison before synergy is claimed. An additional myeloid intervention should then be evaluated against the same combination backbone. Lineage-specific loss and rescue experiments can distinguish essential mediation from correlated recruitment. The mucosal model should also test whether improved distant tumor control persists when epithelial protection is preserved. Otherwise, apparent immune activation may remain inseparable from nonspecific tissue injury.

### 6.2 Define a functional window rather than a fixed schedule

Post-ablation immune events need longitudinal assessment. Nodal antigen accumulation after boiling histotripsy was transient in melanoma (14), whereas the protective neutrophil-clearance circuit operates through a different feedback sequence (6). These findings support serial sampling, not a universal treatment day. Baseline, early antigen-release and later resolution samples could distinguish successful priming from persistent myeloid suppression. Acoustic exposure, treated fraction and thermal contribution should be reported with the biological measurements.

For an initial clinical proof-of-concept, the most informative population would be MSS/pMMR CRC with a lesion amenable to focused ultrasound; colorectal liver metastases provide a clinically studied HIFU target (31), although those studies do not establish mechanical HIFU immunobiology or checkpoint synergy. A testable sequence is mechanical HIFU followed by PD-1/PD-L1 blockade during the early post-ablation priming window, with the interval compared prospectively rather than fixed because the optimal timing is unknown. Antitumor and mucosal-safety assessments should proceed in parallel, including cDC1 peptide-MHC-I cross-presentation, DC-derived IL-12, antigen-specific CD8+ T-cell responses, CXCR2-associated suppressive myeloid populations and NETs, and epithelial-barrier injury; these remain candidate research endpoints rather than validated clinical biomarkers.

Sampling should preserve spatial identity. Tumor-core debris, the viable tumor margin, adjacent epithelium and draining nodes represent different biological compartments. Human CRC spatial profiling demonstrates why bulk averages can obscure organized immune interactions (29). Serial tissue-resolved measurements would therefore be more informative than a single circulating cytokine value. Their purpose is to locate the limiting interaction, not merely to construct a larger inflammatory signature.

As shown in **Figure 2**, a functional sequence can connect antigen delivery, DC licensing and checkpoint-sensitive T-cell activity while maintaining a parallel barrier-safety assessment. This approach remains a testable framework. Direct evidence that neutrophil–macrophage reprogramming improves mechanical HIFU plus PD-1/PD-L1 blockade in human mucosal CRC is still lacking. Prospective studies must establish this causal link before biomarker-guided treatment can be recommended.

**Figure 2:**
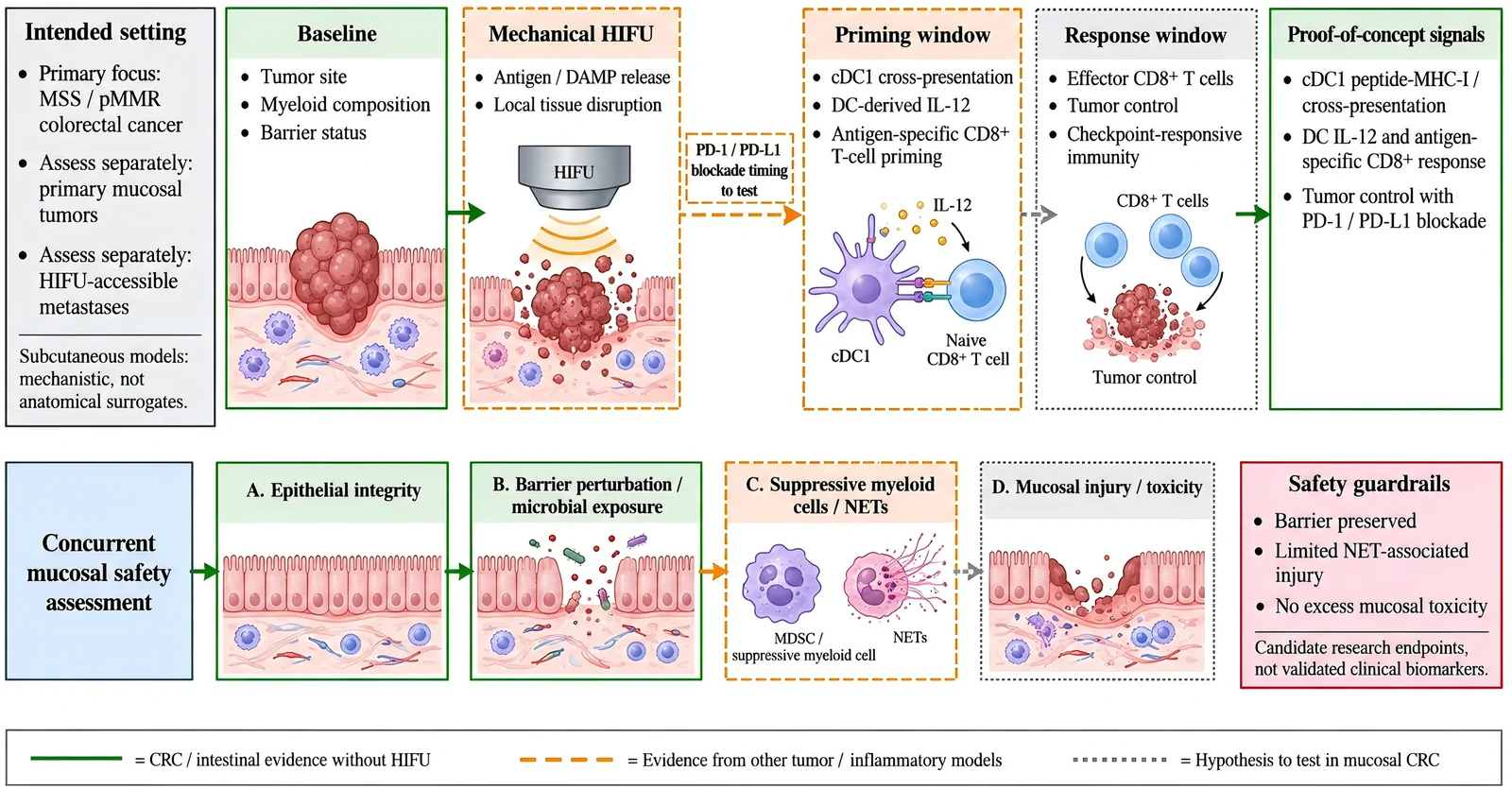
Translational roadmap for testing mechanical HIFU plus PD-1/PD-L1 blockade in colorectal cancer with concurrent mucosal-safety assessment.

## 7 Conclusion

Mechanical HIFU can generate antigenic material, but productive immunity requires coordinated myeloid processing. Neutrophils and macrophages regulate both mucosal protection and the conditions supporting DC–T-cell cooperation. These functions cannot be reduced to uniformly suppressive or inflammatory phenotypes. Integrating antigen-specific priming with granulocyte–phagocyte feedback and epithelial safety provides a rational basis for evaluating PD-1/PD-L1 combinations in CRC. Translation depends on demonstrating this coordination in the intestinal tumor compartment rather than assuming that local ablation creates systemic immunity.
